# Ten simple rules for creating reusable pathway models for computational analysis and visualization

**DOI:** 10.1371/journal.pcbi.1009226

**Published:** 2021-08-19

**Authors:** Kristina Hanspers, Martina Kutmon, Susan L. Coort, Daniela Digles, Lauren J. Dupuis, Friederike Ehrhart, Finterly Hu, Elisson N. Lopes, Marvin Martens, Nhung Pham, Woosub Shin, Denise N. Slenter, Andra Waagmeester, Egon L. Willighagen, Laurent A. Winckers, Chris T. Evelo, Alexander R. Pico

**Affiliations:** 1 Institute of Data Science and Biotechnology, Gladstone Institutes, San Francisco, California, United States of America; 2 Department of Bioinformatics—BiGCaT, NUTRIM, Maastricht University, Maastricht, the Netherlands; 3 Maastricht Centre for Systems Biology (MaCSBio), Maastricht University, Maastricht, the Netherlands; 4 Department of Pharmaceutical Sciences, Division of Pharmaceutical Sciences, University of Vienna, Vienna, Austria; 5 Instituto de Ciencias Biologicas, Departamento de Bioquimica e Imunologia, Universidade Federal de Minas Gerais, Belo Horizonte, Brazil; 6 Micelio, Antwerp, Belgium; Dassault Systemes BIOVIA, UNITED STATES

## Introduction

Pathway models are an effective way to capture and share our current understanding of biological processes. A pathway model is defined here as a set of interactions among biological entities (e.g., proteins and metabolites) relevant to a particular context, curated and organized to illustrate a particular process. Properly modeled pathways can be used in the analysis and visualization of diverse types of omics and other biomedical data [1,2]. The modeling process involves taking our knowledge about biological pathways—however messy and incomplete—and encoding it in standardized data formats that can be shared, reused, and synthesized with other knowledge in accordance with the Findable, Accessible, Interoperable, and Reusable (FAIR) principles [3]. The rules presented here serve as an introduction and guide to the pathway modeling process, leveraging freely available tools and resources.

Biological pathway information is often conveyed as published figures, and rules for better figures, in general, are also relevant when producing pathway models [4]. However, pathway models are more than just figures. In addition to providing an intuitive depiction of a biological process that is easy to understand for humans, they also provide relevant annotations and metadata to be processed by computers. Similarly, rules for network visualizations can be applied to pathway models [5], but the distinct context, layout, and usage of pathway models necessitate specific guidelines and rules. Pathway models are described with specific languages, such as the Systems Biology Graphical Notation (SBGN) [6], the Systems Biology Markup Language (SBML) [7], the Biological Pathway Exchange (BioPAX) format [8], the Graphical Pathway Markup Language (GPML) [9], and many more.

Here, we describe a set of rules for constructing pathway models to optimize their use both as graphical representations for human consumption and as FAIR resources for computational analysis. The rules range from reusability and dissemination (Rules 1, 9, and 10), intuitive visual concepts (Rules 2, 7, and 8), to enabling computational analysis (Rules 3 to 6). We hope that these rules provide a simple framework for pathway model curators who want to create (re)usable resources for the scientific community.

## Rule 1: If possible, reuse and extend existing models

When constructing a pathway model, first, research existing content from the myriad of online databases containing pathway-related information. Searchable databases for biological pathways include Reactome [10], WikiPathways [11], BioCyc [12], KEGG [13], PharmGKB [14], cBioPortal [15], PANTHER [16], ConsensusPathDB [17], Pathway Figure OCR [18], and Pathway Commons [19]. The pathway models from these databases can be used as a source material to be cited or to help build your pathway modeling project. The WikiPathways database is unique in supporting direct community participation, allowing researchers to make edits, extensions, and new versions of existing content using PathVisio [9]. Each pathway model database provides a different angle or abstraction of the existing knowledge on a given topic, relying on distinct approaches to knowledge collection and curation represented by distinct data formats. For example, the citric acid (tricarboxylic acid (TCA)) cycle pathway content was found to be scattered over 5 different pathway databases [20]. The largest source of pathway information by far is scientific literature, where pathway figures provide critical summaries and conceptual models for primary discoveries, which are often the starting point for pathway curation efforts. The Pathway Figure OCR database project has extracted entities from tens of thousands of pathway figures published over the past 25 years and now enables simple search by standardized gene symbols, diseases, and keywords [18]. Tools and resources are being developed to access this content (https://gladstone-bioinformatics.shinyapps.io/shiny-25years) and to facilitate curation by providing starter pathway models with extracted gene sets and by prioritizing pathway figures based on uniqueness against existing pathway databases.

Other valuable sources of pathway information are network and interaction databases such as BioModels [21], NDEx [22], Rhea [23], IntAct [24], Complex Portal [25], and STRING [26]. Some of these databases like NDEx contain context-specific networks, while others are collections of binary interactions (e.g., Rhea and STRING). Most of these databases support searching for more than one entity at a time, as well as by keywords. Ontologies and gene set collections such as Gene Ontology [27] and MSigDB [28] can also be useful in researching the components of a pathway. Additionally, individual entities can be looked up in gene, protein, and chemical databases such as Ensembl [29], UniProt [30], and ChEBI [31], which collect relevant annotations and links to higher-order resources.

By researching these sources, one not only collects supporting evidence for their pathway model but also compiles a more complete and original model. All sources should be cited in the pathway model as part of the modeling process (see Rule 5). There are a variety of free tools available for pathway modeling, including PathVisio [9], CellDesigner [32], Newt [33], SBGN-ED [34], and yED+ySBGN [35]. Many of these are able to import and export existing models in standard formats (SBGN, SBML, GPML, and BioPAX), allowing curators to adapt and extend existing models [36].

## Rule 2: Determine the correct scope and level of detail for the pathway model

**Fig 1:**
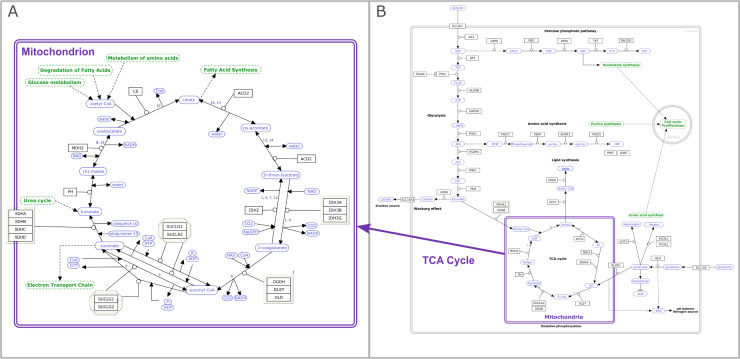
Level of detail/abstraction. The citric acid cycle (TCA cycle or Krebs cycle) represented as the canonical version (panel A, wikipathways:WP78) [37] and as part of processes involved in metabolic reprogramming in colon cancer (panel B, wikipathways:WP4290) [38]. In the canonical model, each enzymatic step in the cycle is represented in detail, whereas when the same process is represented in the context of colon cancer, specific steps in the cycle are summarized and abstracted at a higher level. Pathway nodes are depicted in green, metabolites in blue, and genes and proteins in black. TCA, tricarboxylic acid.

To illustrate a particular biological process, pathway models contain entities and their interactions relevant to the particular context. Thus, the scope of the pathway model, i.e., the entities and boundaries we choose, should be based on what we are trying to illustrate. When deciding on the scope of a pathway model, consider which reactions and entities are crucial to understanding the relevant process. As an example, comparing 2 “citric acid cycle” pathway models at WikiPathways, the canonical pathway [37] has a high level of detail describing the enzymatic reactions critical to this pathway, with links to related pathways represented as pathway nodes (Fig 1A). As a comparison, the “metabolic reprogramming in colon cancer” pathway [38] includes a version of the citric acid cycle where individual steps are summarized at a higher level, but where the full pathway includes other relevant metabolic processes (Fig 1B). For metabolic conversions, one option might be to only include the main reaction participants to reduce visual clutter, thereby omitting proton and electron donors or acceptors (usually relatively small molecules) and ignoring stoichiometry. Similarly, in a cancer pathway model where genes in a central signaling cascade are mutated, the central signaling should be illustrated in detail. On the other hand, if ligand binding processes are the most relevant, the downstream signaling events can be condensed and illustrated at a higher level or even using pathway nodes instead of genes and interactions.

Many pathways reference or include (parts of) other pathways. If the inclusion of another pathway is not central to understanding the process, pathways or parts of pathways should be represented by a single pathway node instead of including many peripheral gene products or proteins. If a more detailed model representing part of the pathway is available, we encourage curators to add links to those pathways in their model. The number of nodes included in a model affects its usefulness in downstream analysis and data visualization. For enrichment analysis, pathway size affects the performance and interpretability indicating that meta pathways or other large pathways describing multiple processes should be split into individual smaller pathways for these types of analyses [39]. Smaller models can be merged for topological analysis and other network analysis approaches where larger networks perform well. For visualization, the size depends on the application and purpose of the visualization (providing an overview or showing a specific detail) and other factors impacting interpretability (see Rule 7).

## Rule 3: Use standardized naming conventions and identifiers for molecular entities

Understanding biological processes and molecular entities can be a difficult job with the vast amount of synonyms in use. For example, the official gene name of neuroepithelial cell–transforming gene 1 protein (NET1) is also a synonym for the sodium-dependent noradrenaline transporter (SLC6A2). Also, well-known chemicals are commonly referred to by multiple names, for example, paracetamol/acetaminophen, which has well over 500 drug vendor–specific names worldwide [40]. Initiatives such as the HUGO Gene Nomenclature Committee (HGNC) [41] and the Mouse Genome Informatics (MGI) database [42] aim to provide a consistent vocabulary for gene symbols and names in such a way that they are unique and do not use problematic characters, are not autocorrected by tools like Microsoft Excel, are not common words, and are intuitively readable. If existing, species-specific common names and gene symbols should be used. If relevant, the synonym or alternative name can be added as a note or comment to the entity in the pathway model.

Names are often more descriptive and understandable to researchers with knowledge of the context; however, this is not trivial for computers. In pathway models, this problem is in part overcome by annotating molecular entities with both a human-readable label and a computer-readable identifier. These resolvable identifiers connect to online knowledge bases containing information about the individual molecular entities. Many of the pathway curation tools have integrated identifier resolution, and curators only need to define the identifier and database for the entity. Given the large number of online databases, choosing the proper identifier for a specific entity can be difficult. It is important to use the most precise identifier with the biological entity. For example, use UniProt for a specific protein, instead of the gene identifier, or RefSeq [43] if a specific protein sequence is meant. For genes, use Ensembl or NCBI gene identifiers [44], which are regularly updated by genome assembly and annotation projects. For species not covered by these 2 sources or where full genomes are not (completely) known yet, gene identifiers from other assembly databases can be used. For resolvability purposes, we advise to register these databases in identifiers.org [45]. For specific gene products, like microRNAs, identifiers from more targeted databases can be used (e.g., miRBase) [46]. Similarly, use ChEBI or Wikidata [47] for chemical compounds, and for lipids, LIPID MAPS [48] or SwissLipids [49]. Annotating groups of proteins acting together or in parallel can be increasingly difficult when the number of individual proteins is large. The Complex Portal [25] provides identifiers for protein complexes, which also enables the possibility to (automatically) check for the participants in the complex. In contrast, gene products acting in parallel (e.g., isoenzymes and assembly factors) that are not directly part of a complex can often be combined into groups. For protein families, InterPro [50] or Enzyme Commission (EC) codes [51] can be used, but only if no other details can be found in the literature. By explicitly stating if proteins belong to either a complex (indicating each subunit is needed to perform an action) or group (meaning each protein alone can perform the same action), the understanding of a pathway model and the possibility for analysis will improve.

In general, consistency in labeling and annotation with identifiers is essential for users and analysis tools to interpret molecular pathway models. This is in line with the FAIR principles [3], which were established to improve the usefulness of data sources for humans and systems.

## Rule 4: Use standardized interaction types

Pathway models need detailed interactions to describe how different biological entities influence each other. Therefore, specifying the meaning of interactions or the relationships between entities constitutes a core task in generating a pathway model. Pathway models use various line styles (e.g., arrows and T bars) to show which biological entities are interacting and how. Although the machine readability of pathway models is made possible by connecting entities, simply connecting entities is not enough. The lines and arrows in diagrams can have several biological meanings, e.g., metabolic conversion, stimulation, and modification, which should be easily distinguishable from one another. This warrants the necessity of a standardized set of biological interaction types used within a pathway model.

Several established standards describe different types of biological interactions, all with a specific focus, for example, SBGN or Molecular Interaction Maps (MIMs) [52]. We advise curators to select and stick to one of the available drawing standards within a pathway model. Additionally, ontologies like the Systems Biology Ontology (SBO) [53], BioPAX, Biological Expression Language (BEL) [54], or Molecular Interactions Controlled Vocabulary (PSI-MI) [55] can be used to further specify and annotate the interactions. Distinguishing between visual interaction types (different arrowheads) and annotation details is important for users to understand the individual biological meanings at first glance. As an example, drawing standards usually have different styles for stimulation, catalysis, and necessary stimulation (MIM and SBGN), while ontologies like SBO could provide further details (e.g., necessary versus absolute stimulation).

By using a standardized set of interactions, pathway authors can specify the correct biological semantics for the interactions in the pathway model. Detailed annotation (type, evidence, provenance, and identifier) of interactions facilitate their use to integrate pathway models with other resources [56]. Mappings between different standards and vocabularies will then allow harmonization between pathway models from different resources or with different drawing styles to efficiently combine and integrate pathway models in computational analysis such as drug discovery [57]. To facilitate the use of such standardized interaction types, pathway tools often include them predefined in drawing panels. Whenever possible, adding identifiers to interactions further improves the identification for analysis purposes. As an example, the Rhea database provides stoichiometric and balanced reactions for metabolic conversion interactions, which is key for pharmacodynamic and kinetic data modelers. Adding Rhea identifiers to interactions also moves the pathway models a step closer to kinetic modeling.

Finally, if you decide to use nonstandard interaction types, always define their meaning in the pathway model in a visual legend.

## Rule 5: Provide literature references, provenance, and evidence for pathway content

Information added to a pathway model, such as proteins or interactions, should be based on scientific evidence or at least a documented hypothesis. The origin of that information, also called provenance, and an evaluation of the evidence should be referenced such that people exploring the pathway model can easily access and verify the source of the information. Curators often add focused references for specific biological entities (e.g., publication about the role of a mutated protein in a pathway) or interactions (e.g., evidence for the inhibition of protein A by protein B). Additionally, many tools allow adding general literature references important for the understanding of the pathway model as a whole. In addition to the reference itself, by using the Evidence and Conclusion Ontology (ECO) [58], one can provide additional information about the certainty of the information, for example, when a pathway or interaction has been inferred from another species.

When provenance is provided in a machine-readable manner, this information can be used to automatically filter pathway content by the presence and type of supporting evidence. Furthermore, the coverage of a pathway model and pathway database can be assessed with more ease. Finally, linking the pathway model content to other databases and retrieving additional information thereof can be done effortlessly.

## Rule 6: Annotate pathway models with a title, a description, and ontology terms

To improve readability and reusability, accurately describing the pathway content on different levels is crucial. First, a descriptive and clear title is important to capture the overarching concept of the pathway model. The title should be concise and limited to a single line when possible. In general, abbreviations should be avoided and only included when the process refers to a specific gene or protein. Second, think of the pathway description as a textual representation of the pathway model, describing each main step as well as the overall process and outcome. While it might seem counterintuitive, writing the description before creating the model helps to make sure that the description is a proper representation of the model in the end. The description should be unique; do not simply copy the description from another source (such as the original caption of a figure). Include literature references within your description, whether they refer to specific components in the model or provide the provenance from which the model was adapted (see also Rule 5). Finally, machine-readable metadata about the pathway diagram improves the findability and organization of the pathway models. Using existing ontologies like the Pathway Ontology [59], the Gene Ontology, the Cell Ontology [60], or the Disease ontology [61] provides the specific context to a pathway model.

## Rule 7: Increase readability with graphical annotations and intuitive layout

Illustrating a biological process in a graphical format clearly and legibly is a challenge. The layout of reactions and entities is critical to readability. For most pathway models, a layout with top-to-bottom and left-to-right orientation is recommended. Cellular compartments and other graphical annotations are helpful to further organize the layout and should be labeled for clear identification. In general, labels on nodes describing proteins, gene products, and metabolites should have one consistent font, size, and placement throughout the pathway model. However, using a larger font size helps to highlight and draw attention to nodes of particular importance. To increase readability, the placement of nodes should avoid creating overlapping interactions as much as possible. Additionally, the layout should be optimized to avoid using multiple copies of the same node, unless there is a biological meaning. For example, when illustrating the transport of a protein into a compartment, 2 copies of the same node are necessary to describe the process properly. Similarly, illustrating 2 different complexes composed of an overlapping set of proteins requires multiple copies of the same node, as does illustrating multiple reactions involving the same common reactants, for example, ATP/ADP.

**Fig 2:**
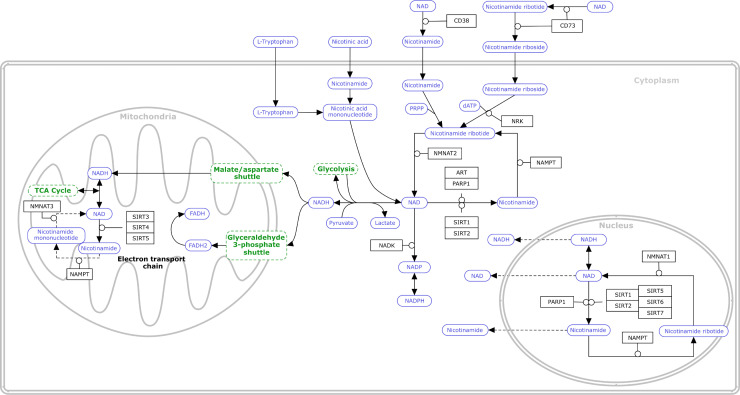
Use of graphical annotations in the NAD+ metabolism pathway at WikiPathways (wikipathways:WP3644) [62]. Since one of the most common uses of pathway models is data visualization on nodes, nodes must be adequately sized to allow for efficient data visualization. Accounting for this, node size can then be optimized to the overall size and complexity of the pathway model, utilizing a smaller node size for models with many nodes and a larger node size for sparse models. A consistent representation of different molecular types in terms of size and shape is important, for example, using a specific shape for gene products/proteins and another for metabolites. Any nonstandard nodes or entities should be defined in a legend in the model, as well as in the textual description. NAD+, nicotinamide adenine dinucleotide.

Fig 2, illustrating nicotinamide adenine dinucleotide (NAD+) metabolism as described at WikiPathways [62], exemplifies these recommendations. The pathway is organized from top to bottom, starting with the entry of precursors (tryptophan, nicotinic acid, nicotinamide, nicotinamide riboside, and nicotinamide ribotide) at the top, using 2 copies of the relevant nodes to illustrate transport. Within the cell, processes are localized to either the cytoplasm, mitochondria, or nucleus.

## Rule 8: Consider data visualization when using colors in your pathway model

Various types of data, like transcriptomics, proteomics, metabolomics, and fluxomics data, can be visualized on pathway models. Whereas data linked to molecular entities (nodes) are often depicted by using a color scheme to fill the nodes, data related to interaction types are often depicted by changing the width or color of the interaction linking the data nodes.

To avoid interfering with such data visualization, the fill color for nodes and edges should be limited when designing your pathway, as they can interfere with data visualization and lead to misinterpretation at the process level. We recommended differentiating molecular entities using node shapes to designate state information (e.g., posttranslational modifications) using dedicated glyphs and to depict different interaction types using standard arrowheads (see Rule 4), instead of using colors. If you decide to use colors in your pathway, consider readability for all users by choosing a visual accessible color palette.

**Fig 3:**
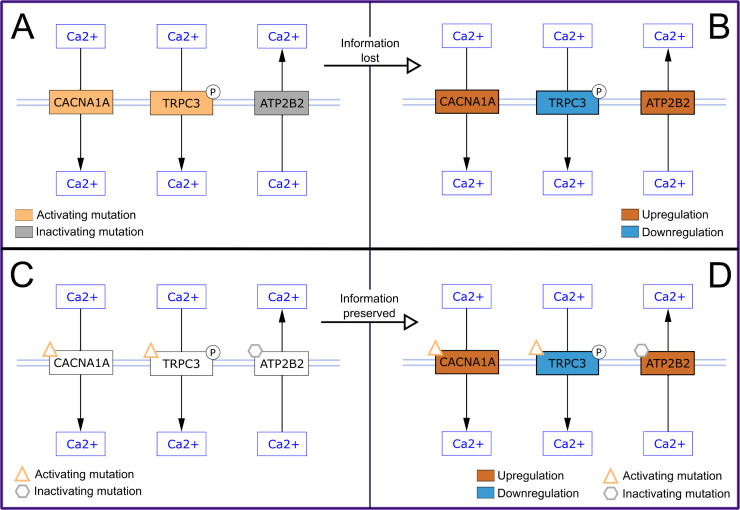
Three visualizations of part of the “PKC-gamma calcium signaling pathway in ataxia” at WikiPathways (wikipathways:WP4760) [63]. Visualizations of part of the “PKC-gamma calcium signaling pathway in ataxia.” **(A)** Node fill color is used to describe genes with mutations relevant to ataxia; orange indicates an activating mutation, and gray indicates an inactivating mutation. **(B)** When experimental data are visualized as node fill color, the mutation information is lost. **(C)** Mutations are shown as an added graphic to nodes; an orange triangle indicates an activating mutation, and a gray hexagon indicates an inactivating mutation. **(D)** Data visualization does not interfere with the mutation information, and both data types are visualized. Pathways were visualized in Cytoscape [64]. PKC, protein kinase C.

In Fig 3A, node fill color was used to describe gene mutations of interest to a disease. When data are visualized as node color (Fig 3B), the fill color related to mutations is overwritten, and the information is lost. Fig 3C illustrates a better solution to illustrating genes with mutations by adding a small graphic to relevant nodes, which is preserved when data are visualized on the node, as shown in Fig 3D.

Finally, when using different colors and line width is unavoidable, include a legend to define the color and line annotations. A legend can assist in differentiating between the data and the pathway entities themselves when visualizing data on the pathway model.

## Rule 9: Communicate and disseminate your pathway model widely

Once your model is ready, you want people to reuse and cite it. With the above rules, you already ensured the easy reuse of the model. But full dissemination still starts with communicating to others that you have created a new pathway model. When your model is being developed as part of a research article, particularly if featured as a figure therein, include at least the machine-readable model as a supporting information. This simplifies the reuse of the knowledge by the readers of your article. You can even take this a step further and make your model available in an open pathway knowledge base, for example, WikiPathways or NDEx, or more general archives like figshare [65] or Zenodo [66]. This also provides a URL, allowing other researchers to find and cite your model easier.

Once you are confident about the content, you can further communicate your work via social media (e.g., LinkedIn [67] and Twitter [68,69]), or, if the model is of wider interest, also in Wikipedia (www.wikipedia.org). You may also be able to include it on your website as an image linking to the machine-readable version or as a compact identifier in text, such as wikipathways:WP78 for WikiPathways WP78 [70]. For pathway models at WikiPathways, an interactive view of the model can be embedded in a web page [71].

As an extension to the World Wide Web, the semantic web is a viable platform to share novel pathway content on. Various pathway resources, for example, WikiPathways, disseminate content in the Resource Description Framework (RDF), which allows rapid reuse of the content in other research workflows that use the semantic web [72]. Network representations of the pathways can be deposited into NDEx, making it accessible directly from network analysis tools such as Cytoscape [64]. The pathway content (gene lists) can also be distributed to multiple enrichment analysis tools, for example, MSigDb. Adding your model to a community-curated resource like WikiPathways automatically ensures broad dissemination of your pathway model in multiple formats and also allows for early review from peers and automated version control during the development of the model.

## Rule 10: Maintain your pathway model as an evolving resource

**Fig 4:**
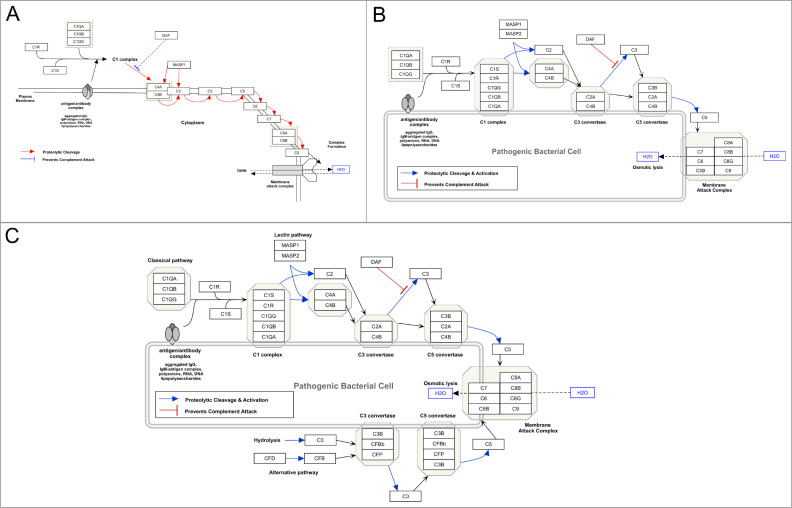
The development of the complement activation pathway model at 3 time points. **(A)** Revision number 63169 as of May 8, 2013 [73]. **(B)** Revision number 82136 as of September 9, 2015 [74]. **(C)** Revision number 106816 as of September 17, 2019 [75].

Pathway models are never finished and should be considered living models, both in terms of content and graphical aspects, as seen in Fig 4. When research reveals new findings or biological insights, these should be added to the pathway model if it fits into the scope of the pathway as originally intended. Just like research, the development, curation, and maintenance of pathway models are community efforts that bring various perspectives and information sources together.

Updates are aimed at improving the graphical layout and content and should be described by the editor in accompanying comments. An example of a pathway timeline is illustrated in Fig 4, showing 3 versions of the complement activation pathway [75] on WikiPathways: (A) The first revision from 2013 is simple, limited to the core molecular entities and interactions and a basic layout. (B) In 2015, the pathway model has been extended in terms of molecular content and their interactions (addition of MASP1/2, details of the complement proteins, etc.), and the layout was updated with the addition of a cell shape, increasing readability. (C) After continuous improvement, the latest revision includes the addition of the alternate pathway as well as the definition of the 2 other subpathways, classical and lectin.

Another reason to keep your pathway updated is the identifier annotation for genes, proteins, metabolites, and other pathway elements (see Rule 3). Databases like Ensembl, ChEBI, and even WikiPathways phase out identifiers in data curation processes, and like broken website links, replacing phased out identifiers in your pathway ensures that links to external databases and general interoperability are preserved over time.

## Conclusions

Pathway diagrams are intuitive tools for collecting and communicating molecular details of biological processes. Every year, thousands of pathway diagrams are published in the literature often as static images, which slow down the distribution of the knowledge, limits reusability, and prevents computational analysis [18].

Pathway diagrams, in general, should be made available, ideally in an online pathway database, as models created with appropriate pathway modeling tools and standards. By following the 10 simple rules described in this paper, pathway models maximize potential for computational analysis and reuse.
